# Integrative modelling for One Health: pattern, process and participation

**DOI:** 10.1098/rstb.2016.0164

**Published:** 2017-06-05

**Authors:** I. Scoones, K. Jones, G. Lo Iacono, D. W. Redding, A. Wilkinson, J. L. N. Wood

**Affiliations:** 1 STEPS Centre, Institute of Development Studies, University of Sussex, Brighton BN1 9RE, UK; 2 Centre for Biodiversity and Environment Research, Department of Genetics, Evolution and Environment, University College London, Gower Street, London WC1E 6BT, UK; 3 Institute of Zoology, Zoological Society of London, Regent's Park, London NW1 4RY, UK; 4 Department of Veterinary Medicine, Disease Dynamics Unit, University of Cambridge, Madingley Road, Cambridge CB3 0ES, UK; 5 Environmental Change, Public Health England, Didcot OX11 0RQ, UK

**Keywords:** zoonoses, modelling, Africa, Lassa fever, Ebola, One Health

## Abstract

This paper argues for an integrative modelling approach for understanding zoonoses disease dynamics, combining process, pattern and participatory models. Each type of modelling provides important insights, but all are limited. Combining these in a ‘3P’ approach offers the opportunity for a productive conversation between modelling efforts, contributing to a ‘One Health’ agenda. The aim is not to come up with a composite model, but seek synergies between perspectives, encouraging cross-disciplinary interactions. We illustrate our argument with cases from Africa, and in particular from our work on Ebola virus and Lassa fever virus. Combining process-based compartmental models with macroecological data offers a spatial perspective on potential disease impacts. However, without insights from the ground, the ‘black box’ of transmission dynamics, so crucial to model assumptions, may not be fully understood. We show how participatory modelling and ethnographic research of Ebola and Lassa fever can reveal social roles, unsafe practices, mobility and movement and temporal changes in livelihoods. Together with longer-term dynamics of change in societies and ecologies, all can be important in explaining disease transmission, and provide important complementary insights to other modelling efforts. An integrative modelling approach therefore can offer help to improve disease control efforts and public health responses.

This article is part of the themed issue ‘One Health for a changing world: zoonoses, ecosystems and human well-being’.

## 1. Introduction

‘One Health’ approaches argue for an integrated, holistic approach. Understanding intersections between disease dynamics, environmental drivers, livelihood systems and veterinary and public health responses is essential. In this paper, we ask how to understand these complex, non-linear, multi-component systems, and the intersection of natural-social systems within these; and in particular, how can modelling help? Looking at Lassa fever and Ebola virus disease and disease dynamics in West Africa in particular, we contrast process, pattern and participatory modelling, and suggest an integrative approach that encourages a conversation between different modelling insights.

Models are ways of understanding the world from different perspectives. They are simplified frameworks for understanding. Models come in all shapes and forms—they can be quantitative or qualitative, inductive or deductive, expert-driven or participatory, closely connected to data, or centred largely on assumptions; they can provide precise predictions, assessments of risk or simply offer heuristic insights. They can be used by different people too. They can provide the basis for decision making, they can be tools for policymakers or simply ways of opening up debates about complex systems among different actors. Importantly, models always ‘frame’ knowledge and policy and have a social and political life, which can be important in disease response.

In this paper we discuss the experience of integrating modelling approaches in the Dynamic Drivers of Disease in Africa programme (http://steps-centre.org/project/drivers_of_disease/). Modelling approaches looked at knowledge about disease, ecosystem and poverty and livelihood interactions from different perspectives. Some models have examined the *processes* of disease spillover, based on a mechanistic understanding of the system. Others have looked at *patterns* of disease incidence and impacts, based on statistical analysis of macroecological and social patterns over space, using geographical information systems. Others develop more bottom-up understandings of both patterns and processes from analysis, involving *participation* of those who live with disease, and who are embedded in the socio-ecologies of concern.

Rather than attempting to build ever more complex models, we instead make the case for an approach to integrative modelling that asks questions and facilitates conversations about complex disease, ecosystem and livelihood interactions from different perspectives, with different model assumptions and data sources. This is centred on combining three Ps—process, pattern and participation—in modelling efforts. In the next section, we outline these three diverse approaches, and make the case for their combination.

## 2. Modelling zoonoses

### (a) Process-based modelling

Process-based models are theoretical representations of the biological mechanisms of interest and sometimes their physical drivers [1]. These models can be built on first principles or on functions that describe some of the relevant processes. An important class of these models, but not discussed here, is based on numerical simulations, such as agent-based models, that mimic the biological processes with a computationally aided set of autonomous, interacting agents [2]. Here, we focus on population dynamics models, which are typically used to calculate changes, such as size and age composition, in the populations of interest. In compartmental versions, the population is partitioned into relevant epidemiological categories, such as susceptible (S), exposed (E), infected (I), and recovered or removed (R) individuals [3]. The roots of these models can be traced back to the beginning of the twentieth century [4,5], and they have been widely extended; for example through including stochastic effects [6], spatial variability, heterogeneity in the network of contacts [7], multiple species [8], age-specific sub-populations, evolutionary dynamics [9–11] and incorporation of environmental components [12].

The underlying assumption of many compartmental models based on a set of differential equations [13] is that the population is large; even some stochastic versions, such as those based on van Kampen/Kramer Moyale expansion [11,14], rely on this assumption. This class of model may be appropriate to study the infection dynamics in the reservoir host, with large populations, but their suitability is questionable for the study of infrequent zoonotic spillovers. There are important exceptions, for example compartmental household models based on Poisson and/or branching processes [15,16], stochastic compartmental models based on the solution (either exact or approximate) of the Master equation [17], and particularly popular are stochastic compartmental models employing the Gillespie algorithm ([6,18] and references therein). A major challenge is that stochastic models require a large number of replicate simulations to establish confidence in results, especially when dealing with rare events such as zoonotic spillover. Furthermore, spillovers are often caused by complex interactions of multiple causes, including ecological factors (e.g. presence of hosts with differing degrees of susceptibility and periodicity in their abundance), epidemiological and genetic factors (e.g. a broad set of pathogen life histories and periodicity of infection prevalence), and anthropogenic activities (e.g. a land-use and behavioural changes affecting direct and indirect interactions with reservoir hosts). Not surprisingly, theoretical [18–22] and experimental studies able to disentangle the many complex aspects of transmission at the animal-human interface are scarce [23,24]. Recent developments include incorporation of branching processes [25,26], Hawkes processes [27] and binomial processes, coupled with pattern-based, macroecological approaches (see below). Despite the need for a new paradigm integrating biological, social and environmental sciences with mathematical modelling being increasingly recognized [28,29], theoretical frameworks fulfilling this objective are rare.

### (b) Pattern-based modelling

Pattern-based modelling is based around correlations or statistical associations between empirical data. This type of modelling approach is widely used in ecological research to explore associations between species’ characteristics or traits and environmental variables (e.g. temperature, rainfall, habitat, human population density) at large spatial and temporal scales [30–32]. Macroecological research includes the use of environmental variables and evolutionary histories to understand global spatial patterns of species richness across large taxonomic groups (e.g. [33]), the understanding of species responses to climate change (e.g. [34]), and examining trait evolution or diversification across the evolutionary history of particular groups (e.g. [35]). Interest in using pattern-based modelling, with a macroecological approach, to understand wildlife or human pathogen emergence, persistence and spread has been growing over the past decade. Early studies focused on understanding which wildlife host and pathogen traits correlate with pathogen richness across different species [36]. For example, wildlife host traits such as body size, and longevity as well as population level traits such as density, population structure and geographical range size have shown associations with pathogen richness (reviewed in [37]). Understanding which factors influence wildlife host pathogen richness may in turn aid in targeting particular species for disease surveillance, as it is probable that these species have a higher likelihood of pathogen transmission to other species (including humans). More recently, interest has focused on spatial correlates of human as well as wildlife host pathogen richness; for example, strong latitudinal gradients have been found in the richness of human pathogens [38,39]. Globally, human pathogen richness has been shown to correlate positively with the spatial distribution of vertebrate species richness and negatively with healthcare spending [40].

Pattern-based, macroecological modelling has also been applied to understand the disease emergence process itself. One study focused on the spatial and environmental correlates of the initial zoonotic detection event [41]. Here, human population density, reporting effort and mammal species richness were found to correlate spatially with the first detection of zoonotic disease across 335 emerging infectious human diseases from 1940–2005, resulting in the first macro-epidemiological disease risk maps [41]. Other studies have focused on understanding stages further down the emergence process, to identify which particular wildlife groups share more pathogens with humans, highlighting where disease surveillance is needed most. Primates, even-toed ungulates, carnivores and bats have been shown to share the greatest number of pathogens with humans, suggesting that these species should be prioritized for disease surveillance as reservoirs of zoonoses [42,43]. Others have attempted to investigate the specific trait and spatial correlates of reservoir host status—for example primates [44], rodents [45] and bats [46]—to identify particularly risky species or areas.

Pattern-based approaches have also been used to identify correlations between spatially explicit disease case data and a suite of covariates [47,48]. Such approaches can give insight into the underlying causal mechanisms across spatial scales; for instance, rainfall driving Lassa fever outbreaks via changes to reservoir host numbers [49]. One advantage is that such pattern-based spatial models require only limited knowledge of the disease prior to analysis, and so are ideal for investigations into neglected tropical zoonoses. Such an approach can also start an iterative cycle of investigation, whereby a presumed driver—such as rainfall in the Lassa fever example—can be analysed further using detailed experimental or sociological research. Correlative pattern-based modelling can also be used for purely predictive purposes, such as for covariate-based interpolation of risk for poorly known disease systems (e.g. Ebola [50], dengue [51]). Here, existing statistical correlative methodologies such as machine-learning (e.g. MAXENT [52] or Boosted-Regression Trees [53]) fit the best correlative models to the spatially explicit data, and then use these models to predict the presence or risk of a disease across a landscape [54]. Recently, spatially explicit Bayesian hierarchical models are being employed to extend this approach by applying different pattern-based models simultaneously across both space and time, reflecting different processes driving the occurrence of a disease (as for Rift Valley fever [55]).

Using complex correlative models to predict cases of human zoonotic diseases, as in the above examples, assumes that the two-stage process of wildlife to human disease transmission can be adequately captured (i.e. how wildlife reservoir hosts and the environment interact, and also how humans and reservoir hosts interact subsequently). Although each step in the process might be separately accurately approximated in a pattern-based spatial model, this approach is likely to be limiting when modelling more complex interactions, such as those akin to an invasion (e.g. [56]). Additionally, for understanding how disease risk will change in the future, pattern-based approaches may not be a suitable modelling approach, as the stability of the inferred underlying processes cannot be assumed [54]. This is why linking pattern-based, correlative approaches to process-based modelling—for example, seeing disease spillover and transmission as a process of species ‘invasion’ [46]—can be powerful (see below).

### (c) Participatory modelling

Participatory modelling engages local people in exploring relationships between diseases, ecosystems, livelihoods and well-being. Understanding the contexts for disease spillover and transmission requires detailed knowledge of local landscapes and livelihoods, and so involving local people can enhance understandings significantly. For example, participatory epidemiology has been used to explore local disease classifications, to rank diseases and their effects, to examine disease patterns historically and seasonally, to map disease transmission and risk, to explore the relationships between social differences (age, class, gender, ethnicity) and disease, as well as understanding the social, economic and livelihood context for disease [57]. Equally, a suite of participatory and rapid appraisal techniques, including participatory mapping, seasonal calendars, proportional piling, matrix scoring and ranking, network and movement maps, historical timelines, social maps and transect walks can be combined to gain a richer understanding in any setting (e.g. [58,59]). These can be complemented with ethnographic approaches, involving deeper cultural understandings of disease from a local perspective [60]. All of these approaches and techniques can contribute to participatory modelling efforts, where discussions between local people and external experts can facilitate modelling efforts, either as inputs into quantitative models or elaborations of more qualitative analyses.

Social science perspectives, linked to understandings of local livelihoods, have been important in epidemiology from its beginnings, but there have been few attempts to link participatory insights to modelling, despite the growth of quantitative modelling of disease dynamics in recent years [61]. Participatory insights can enhance such modelling efforts in a number of ways [59,62,63]. Most importantly, understanding disease contexts, including social, cultural, political and economic dimensions, can be very important in framing the model enquiry and so structuring a model. Detailed insights into local livelihoods—such as seasonal agricultural practices, settlement patterns or movement to markets and along trading routes—can be important in challenging simplistic, often averaging, assumptions about transmission dynamics in models. Gaining insights into social difference can uncover gendered, age-specific, occupational or ethnically linked effects on disease susceptibility or transmission effects [64]. Many models are data intensive but also rely on databases of highly aggregated data, which fail to differentiate between places, times and different groups of people. However, participatory data can help parameterize models with realistic data from the field or provide realistic adjustments to estimated data downloaded from global databases, and so qualifying modelling outputs in important ways. Rather than trying to make use of highly differentiated and site-specific data in ever more complex models, the aim is to facilitate a conversation between local realities, as understood by those confronting and managing disease on a daily basis, and the necessarily more simple and abstract models, interrogating the robustness of model assumptions, assessing the validity of data inputs and exploring inherent uncertainties in model outputs.

Where quantitative disease modelling has been integrated with participatory analysis, the result has shifted diagnosis and response, sometimes dramatically. Local epidemiological insights can help ‘where there is no data’ [65] to generate important insights into disease prevalence, spread and impacts. Data collected through participatory techniques—for example matrix scoring—proved helpful in reshaping responses by veterinarians and policymakers, for example, in investigations of a chronic wasting condition of cattle in South Sudan [66], as well as of trypanosomiasis in Kenya [67], foot and mouth disease in Ethiopia [68] and contagious caprine pleuropneumonia in Tanzania [69]. Participatory approaches can be used in disease response planning, monitoring and evaluation, as well as in disease searching and identification and modelling. For example, in South Sudan, participatory methods were used to generate basic data for a model of rinderpest [70]. Information on herd composition—age and sex structure—was crucial to the definition of the basic reproductive number (R_0_), while participatory mapping and seasonal calendars helped in defining contact rates between communities and herds across seasons. This assisted in the design of the final surveillance programme of the disease in one of its final refuges [71].

These examples have been in settings in Africa around livestock disease challenges where data limitations are extreme. However, participatory engagement is not just about filling data gaps. In areas where there is a surfeit of data, modelling and response efforts can be substantially improved through engagement with local people and field practitioners, who may be able to share local experiences of risk and in turn challenge model assumptions through local experiential and tacit knowledge [72,73]. Negotiation of uncertainties, which are always present, must involve diverse perspectives, involving the opening up of debate about different assumptions and interpretations [74,75] (cf. [76] for a discussion of the UK foot and mouth epidemic).

## 3. Integrative modelling approaches

**Figure 1.**
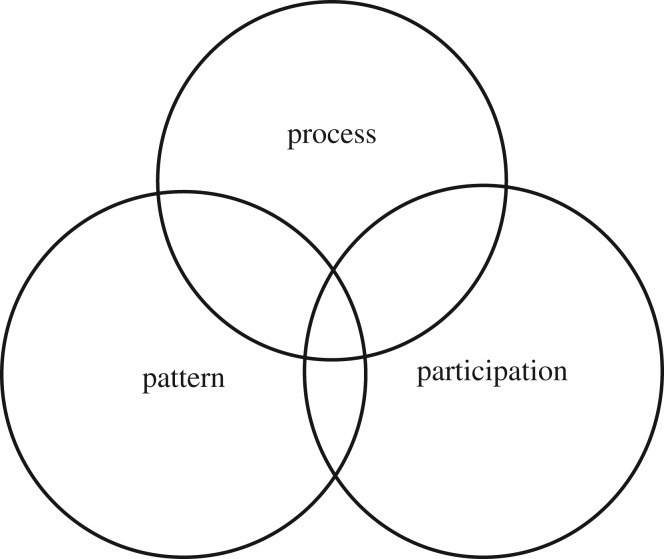
The 3P approach—a conversation between modelling approaches.

There has been a significant growth in the modelling of disease dynamics over the last 30 years. However, this has not necessarily resulted in greater cross-disciplinary integration. A recent review showed how disciplinary silos persisted, with ecological modelling and veterinary medical models remaining distinct [77]. There has though, been a growth of more integrative ‘One Health’ modelling literature, involving epidemiologists, public health specialists and others. However, this shows little engagement with participatory modelling, and social science perspectives. Within the broad One Health field, research remains dominated by veterinary science, centred in relatively few locations, largely in ‘northern’ settings [78]. Thus, despite the repeated calls for cross-disciplinary, inter-sectoral integration, this has been slow to take off. In the following sections, we make the case for integrating modelling approaches in ways that allows a productive conversation between perspectives. The 3P approach—combining process-based, pattern-based and participatory modelling—is offered as a way forward (figure 1).

The aim is not to develop a fully elaborated model for all situations, or to add more and more detail, which limits the generalisability of models; instead it is to enhance literacy around the kinds of limitations and benefits that all models have, and encourage more robust use across disease preparedness, prediction and response. The integration of modelling approaches and the level of detail would depend on the purpose of the model; for example, if it was being used to anticipate disease emergence or to deal with an ongoing epidemic. We therefore do not argue for a grand, synthetic approach; more a platform for informed interaction between perspectives. We contend that this will more likely lead to new insights, retaining the depth and specificity of the individual approaches, but pushing them to articulate with each other, in model design, data parameterization and output analysis. By making explicit the assumptions about any model's framing—whether embedded in model structure, scale or data sources—we can develop a more honest appraisal about their implications for policy and practice. In this way, models can then take on a more transparent social and political role in policymaking. All models have social and political ‘lives’, and many have huge influence, especially in the context of disease responses [79]. Encouraging a more honest debate about models—and the uncertainties within these—through interactions across diverse groups will, we argue, result in a more robust and effective response, which must always be plural and conditional [80].

## 4. Combining modelling approaches: applications to zoonoses in Africa

In this section, we discuss examples of where modelling approaches have been combined. To date, most such linking efforts have occurred within the realm of quantitative disease dynamic modelling; for instance the linking of conventional mechanistic epidemiological models with spatial approaches [24]. Such spatial extensions can, for example, help answer questions about clusters, contact/movement networks or geographical and macroecological factors influencing disease spread and impacts. Thus, human contact networks can be used to construct complex mechanistic models where rates of contact and effective transmission can be estimated and applied to a variety of scenarios to predict how directly transmitted disease may present differently in human populations given certain interventions [81,82]. Such dynamics can be explored further by combining scenario-based simulations of epidemic infections using transport networks alongside simple models of human movement [83]. While extremely detailed mechanistic spatial models of zoonotic disease have been devised for rabies and malaria [84] and have proved useful for making predictions of disease spread [85], these are hugely data-intensive approaches.

A recently developed environmental-mechanistic approach [54] combines correlative species distribution models of host presence with a discrete-time stochastic simulation of subsequent disease cases within human populations, thereby matching the analytical technique to data that are readily available. So far this approach has been adapted to run on simple host–human transmission systems, modelling spillovers across space and time. Such models can also run on more complex transmission systems, incorporating host–human spillover and human-to-human transport networks. In the following two sections, we look at two diseases—Lassa fever virus and Ebola virus disease—and show how a combination of pattern-based macroecological modelling and mechanistic process-based modelling can be a powerful approach for understanding disease risk and transmission dynamics.

### (a) Case 1: Lassa fever models

Previous studies of Lassa fever virus disease have identified the relationships between disease risk and the distribution of the major host, *Mastomys natalensis* [49]. Furthermore, climate, land use and habitats will likely have an impact on host distribution and subsequently disease risk [86]. However, to understand such changes we need to create integrated models of zoonotic spillover and human-to-human transmission.

Two approaches have been used. First, a process-pattern approach. Here a mathematical framework based on a generalization of Hawkes processes [87,88], with inclusion of biological mechanisms, has been formulated [27]. Zoonotic spillovers are assumed to arise from random and independent contacts with the reservoir, with no influence of past infections. In contrast, the number of past human infections affects human-to-human transmission, as each infected person can also trigger a chain of new cases. Both processes are also affected by past events due to depletion of susceptible individuals through death or development of sterilizing immunity. There are many ways such a mechanistic approach could be improved. For instance, spatial variation and explicit effects of environmental drivers (rainfall, vegetation index, etc.) are not included in this model. One way to achieve this would be to use outputs from macroecological models for host infection prevalence and abundance. These parameters could be readily used to estimate the spatio-temporal distribution of the risk of Lassa fever, and determine how environmental drivers have an impact on different modes of transmission.

Second, a pattern-process approach is possible. This involves a different modelling approach that more explicitly links Lassa fever virus spillover to climate change, human population growth and land use [54]. This environmental-mechanistic modelling framework allows the incorporation of future predicted environmental change in order to understand the impact of global change on disease cases [54]. Here, across fine-scale grid squares covering West Africa, spillover and transmission rates were simulated using a homogeneous, random mixing of infectious reservoir hosts with susceptible human individuals using a gas model. This movement and contact model was weighted by spatially variable weighting factors associated with non-mechanized transport (in turn correlated to Gross National Income, and so specific for each country) to capture spatial differences in host abundance and in human movement patterns linked to poverty incidence. This model predicts an overall increase in Lassa disease cases per year by 2070 within the endemic western Africa region, driven in particular by changes in climate and increases in human populations.

These models are of course subject to important limitations of available data; in particular the lack of information on exposure to the reservoir, the effect of reporting bias, and the assumptions made about movement and mixing between identified, hospitalized cases of disease, who could potentially be in contact with each other. As we argue further below, this is an area where participatory modelling and ethnographic research [79] could address critical data gaps; for example on human–animal interaction, health seeking behaviour and actual patterns of mobility and social networking, and hence potential contact patterns.

### (b) Case 2: Ebola models

The Ebola fever disease outbreak in West Africa has been modelled extensively, both retrospectively and in real time. The initial, most simple compartmental (SIR/SEIR) models projected potentially huge mortalities, and raised the alarm [89,90]. Yet they did not take account of spatial dynamics of disease spillover and spread. Spatial dimensions proved to be crucial to understanding Ebola virus disease's impact in West Africa, with major implications for assessing who is affected and where, and so defining risk and response.

Spatial dimensions subsequently became the subject of modelling efforts, resulting in very different predictions and recommendations, as the importance of movement, cluster and network effects were recognized. Major outbreak response interventions and significant behaviour change, particularly around funerals and burials, changed dynamics in important ways, requiring revisions in model parameters over time [91–94].

As with the Lassa fever model described above, combinations of process-pattern and pattern-process models are possible, allowing predictions of outcomes based on how different compartments across grid squares might change, at national, regional or even global scales, for example. In these models, examining the impacts of movements and so contact rates between spatial units is especially important [95]. Equally, spatial-temporal agent-based models have also been used to look at the effects of non-pharmaceutical interventions, including the availability of treatment units, safe burial procedures and household protection kits [96]. Spatial and temporal analysis was enhanced by genome sequencing and phylogenetic analysis, giving important insights into disease spread [97,98]. Meanwhile, compartment models have been extended to look at the network effects of mixing of people within and between communities [99].

Both these disease cases illustrate why single models are insufficient to understand complex, dynamic processes, and why combining process and pattern modelling approaches has real benefits. For example, disease dynamics modelling has been enhanced through bringing in a spatial dimension to standard epidemiological approach based on compartment-based mechanistic modelling, as highlighted by the environmental-mechanistic approach. However, such models usually offer insights at a particular scale, based on available macro spatial data, and this may not relate to spatial patterns that are important on the ground. Models also necessarily make significant assumptions about key transmission parameters, based on available data. Thus, for example, estimates of movement, and so contact, may rely on some major assumptions about people's behaviour. Increased computing power makes simulations over many years and across multiple grid squares across the whole world feasible, but, as with any such modelling effort, the outputs are only as good as the assumptions made and the data supplied.

While unquestionably such work has improved understandings and posed important new questions, we must also ask, are such combined models able to offer sufficient insights that articulate with local needs and understandings, at an appropriate (often quite micro) spatial scale? Do they help us unpack the big ‘black box’ of transmission dynamics in appropriately nuanced ways to help target interventions? In the next section, we therefore ask: how can model structure and data input be improved through other insights, based on more participatory approaches, which are focused on a scale more coincident with patterns of human interaction and disease transmission?

## 5. Perspectives from the ground: a missing element in disease modelling?

In this section, we argue that combining process and pattern modelling approaches with participatory modelling is crucial, as this is a vital missing element in current efforts. Both communicable and non-communicable diseases frequently occur in clusters or along social gradients, affecting particular groups of people or social strata. Epidemiologists have increasingly sophisticated techniques to decipher these patterns, yet when it comes to modelling infectious disease emergence and epidemics, the aggregate data used in many models implies that all people face and pose uniform risks of infection. However, by understanding more about the heterogeneous dynamics of transmission, we can improve both the structure of models and enrich the data that goes into these. Often, there is much local knowledge about socially differentiated exposure that could be integrated into disease modelling efforts. This local knowledge does not just provide more detailed local-level data to make models more comprehensive, it can challenge assumptions of model structure that may need to be revised.

Indeed, with Ebola spreading on a scale that had never been known before, and amidst real uncertainty about how to respond, a turning point in the West African epidemic was when villagers ‘learned to think like epidemiologists’ and epidemiologists ‘learned to think like villagers’ [100,101]. This productive meeting of different perspectives on disease-risk interactions enabled previously obscure things to be seen. Understandings of funeral practices and the risks associated with them was a key area where simplistic modelling predictions improved over time. For example, instead of seeing funerals as discrete events that could be easily modelled and targeted for disease control, a broader set of socio-political arrangements and their implications came to be recognized (see below). By involving more participants in model co-construction, models become more robust and effective, but also more legitimate and transparent in charged policy contexts. In the following sections, we explore the cases of Ebola and Lassa fever further to show how participatory and ethnographic insights are a powerful tool. We offer five simple questions—who, what, where, when and why—that can help to unpack the black box of disease transmission in many models.

### (a) Social roles and relations: who?

Social roles and responsibilities often shape disease transmission. As a social disease that spreads when people care for the sick and the dead, this was especially acute for Ebola. Understanding who takes on caring roles, and who receives what kind of care, is key. Although not exclusively a female activity, women tend to perform home nursing roles in the Mano River region. At the height of Ebola's rampage through Monrovia, women reported that it was inconceivable that they would not tend to a sick loved one [101]. With treatment options extremely limited in August 2014, they chose likely death over abandoning a family member. Men were initially assumed to be more protected, yet this overlooked their obligations in carrying the sick (in hammocks or on motorbikes), preparing graves and conducting aspects of burials.

Social status can influence exposure. For Ebola (and conceivably for Lassa fever too) sickness and death among authority figures—such as healers, male and female society heads, or chiefs—posed greater risks [102]. As renowned members of their communities, news of their illnesses would bring larger numbers of people than normal to come to pay their respects, both before and after death. Because Ebola victims are most contagious just before and after death, this increased rate of visitors and contact earned these high status individuals the label ‘super-spreaders’. Super-spreading dynamics caused critical but uneven boosts to transmission; for example, up to 300 cases of Ebola were traced back to the death of one traditional healer in Sierra Leone [102,103]. Each disease will differ, but the implication is that transmission pathways will be distinct for different social groups, and not random nor easily averaged or spatially defined. This may be more important for non-airborne diseases such as Ebola and Lassa fever, where bodily contact is required to transfer the virus reliably. Taking such heterogeneities seriously during the construction, fitting and validation of models is potentially invaluable, particularly when trying to capture unexplained variance in well-defined datasets. Care would be needed to ensure that models do not become too complex and hence fragile, especially when data are limited and extrapolation from the data is a central aim.

### (b) Unsafe practices: what?

Disease predictions and control are greatly helped by accurate knowledge of what behaviours are driving disease. Aspects of behaviour are already included in disease models at macro levels, such as the impact of non-mechanised transport [54] or funerals. Attention to behaviour is also central to field epidemiology and the public health interventions built on it. However, without being open to the insights of local people and their perspectives, there is much scope for misunderstanding. This can lead to certain practices being blamed and others overlooked—often with detrimental effects.

For example, in the second half of 2014, Ebola was surging and attention was drawn to the absence of safe burials and treatment facilities; especially worrisome for officials was sustained and increasing transmission in the community and the effect of funerals. Left unchecked, models predicted millions of cases [89]. The public health response was to build treatment facilities, ban ‘traditional funerals’, impose blanket ‘safe’ burial policies and impose quarantines. These were implemented poorly initially, and added to problems [104,105]. For example, it was not clear what aspect of funerals—a multi-faceted social process lasting a number of days and involving different sets of people and obligations—a ban was supposed to be targeting. Without making provisions for alternative arrangements across these different dimensions of funerals, ‘safe burial’ procedures ran up against resistance, and at times, drove the disease underground until policies that were more sensitive to the concerns of extended families and local communities were developed. In Central and East Africa local communities have strategies for dealing with Ebola that are biomedically effective despite being based on non-biomedical concepts. For example, *gemo*, the local concept of epidemic disease, is caused by spirits but local response protocols involve isolation, restricted movement and survivors providing care [106]. Building on pluralistic healthcare traditions in the Mano River region, the more successful interventions were those that adapted funeral procedures to accommodate spiritual, economic and biomedical concerns; for example carrying out reparation ceremonies when usual burial procedures could not be carried out because of safety concerns [107].

In the case of Lassa fever most public health messaging and research has focused on the consumption of rodents and hygiene around the home. Messages to keep homes clean and food covered fail to engage with the realities of people's lives where mud floors in their houses are perfect for rodents to burrow, where spare containers to store rice or cooked food are hard to come by and asking people not to keep food in their homes is fanciful. Focus on behaviour is necessary but in order for it to be helpful and to enable the prediction or reduction of vulnerability it must be accurate and sensitive to local conditions, rather than blame, cause resentment, or worse, drive practices that further the spread of disease.

Building a spatially and temporally sensitive modelling approach to Ebola spread requires close attention to the sites of spread, and the social practices involved. Fuzzy boundaries, complex social connections and socially defined, but shifting, practices all affect disease dynamics, often in quite fundamental ways. Gaining insights from local contexts is thus essential, especially when considering appropriate interventions.

### (c) Mobility and movement: where?

Centuries of war, farming and trade mean the Mano River area most affected by Ebola is made up of nested settlements—villages, satellite villages, headquarter towns, abandoned settlements, farm huts, market towns and increasingly larger urban areas—linked by roads and bush paths. These intersect with hierarchies based on slave and founder relationships, and kinship ties. Cross-border movements are common between ethnic groups spread across the three affected countries, reinforced by recent refugee movements. In Sierra Leone, if a man dies away from their home it is common to repatriate their body in order to maintain the land rights of their descendants, while women's bodies are often returned to their villages of birth if their bride-price is not fully paid [100]. People travel great distances on motorbikes, and in shared taxis, but also in hammocks or on foot, including when they are sick. Improved connecting roads and expanding urban areas also shaped the way Ebola spread. The relationships affecting movement, many of which are not immediately obvious to outsiders, and indeed are often deliberately obscured [108], mean both people and viruses do not move about randomly, as assumed in many models.

For Ebola, ‘pulse’ dynamics were observed: a cluster of people would get infected in market towns or trading points—which were often where pharmacists and dispensers were based—and these people would then travel back to their villages, where the epidemic spread more quietly and along bush paths, only to surface again via health-seeking at trading points [109]. In the denser urban areas and slums of Monrovia and Freetown it spread more ferociously and evenly. Understanding the social and political dimensions of mobility is therefore critical for any modelling effort. Such deep knowledge of local ties, relationships and movement patterns is invaluable for contact-tracers, but it also adds another layer of sophistication to models attempting to understand the impact of travel on disease spread, be it through road networks or the availability of transport. Simple algorithms or standardized data may actually result in misleading results from models that do not take this into account.

### (d) Temporal change: when?

The way seasonal changes influence disease spread is central. This goes beyond levels of rainfall or humidity, which can easily be incorporated into models through available databases. Seasonal patterns of human behaviour, especially in agricultural cycles, can be crucial, and this requires deeper understanding of local contexts. In Sierra Leone, reported Lassa fever incidence peaks in the dry season (February-March) [110]. Our research found that rodents were most abundant during this time period, especially in swamps and cleared farm land [111]. Crucially, this is when people are doing intensive hands-on work: for men, felling trees and brushing land, which is likely to displace burrowing rodents; and for women, building and tending to vegetable mounds in swamps. Seasonality is therefore also gendered, with major implications for patterns of disease exposure [64], and so model design.

As diseases spread within an area, different patterns of infectivity may result as people learn and respond. This was important for Ebola and changed the course of the disease. But early models did not take this into account, predicting instead an exponential growth in infection, offset only by mortality. Temporal patterns of infectivity are also important. With Ebola, an infected person is most contagious just before and after death when their viral load is at its highest [112], meaning that sensitivity in models to when people are infectious, and what contacts they have at this time, is crucial. People's behaviours change for many reasons. For example, the scale of the horror in Monrovia, or the impact of high-profile health worker deaths and riots in Kenema, have been credited with shifting views. Such complex and non-linear learning processes are likely to be too unpredictable to model but have a pivotal impact on transmission dynamics, and need to be taken into account.

### (e) Long-term dynamics: why?

What makes a region vulnerable to disease? Many models suggest answers based on identifying key ‘drivers’. However, these are constrained inevitably by what parameters are included, and what data is available. Most models of Ebola and Lassa, for example, point to economic factors (levels of income and poverty), geography (influencing spillover probability and road connectivity for example; see discussion above, and [54] as a specific example) and health service infrastructure [113]. However, for understanding disease emergence and epidemic risks this analysis is often insufficient. Long-term patterns of inequality and insecurity are embedded in social relations and institutions—from slavery, to conflict and war, to mining extraction and modern forms of private sector-dependent development [114,115]. West Africa's Upper Guinea Forest carries an anthropogenic forest-farm landscape that has emerged through centuries of settlement, farming and trade [116] in which people, bats, rodents and other wildlife have long co-habited. In different places, because of particular political ecologies, we must ask whether spillover is recent, the result of new disturbances, or whether an artefact of improved detection [117]. All these dimensions therefore create particular, socially differentiated and location-specific forms of vulnerability, generating both precarious livelihoods and often distrustful publics.

Therefore, models that focus only on short-term drivers and proximate factors, and do not take account of longer-term dynamics and deeper social and ecological histories may fall short. For this reason, a historical understanding of social and ecological change of an area, based on local insights and understandings, can be essential in framing modelling efforts, lest key dynamics are missed. We do not consider here the validity and use of modelling specific elements of epidemics or healthcare; for example, models considering the utilization of healthcare facilities during epidemics can be an invaluable aid to planning and mitigation.

Combining process, pattern and participatory modelling is not easy, and certainly in our collective work we only got so far. Effective integrative modelling requires iterative, long-term, collaborative work, moving from abstract modelling to the field and back and forth across multiple conversations to build and deepen understandings. This takes time, trust and patience, and may well involve ‘constructive conflict’ of different sorts [118]. It requires spaces for the conversations between modelling processes to be opened up, and for some letting go of languages, approaches and disciplinary and professional strictures. Such engagements must not just involve researchers, but an effective transdisciplinary engagement must involve local people, government officials, veterinarians, doctors and diverse practitioners involved in the dialogue that frames, designs, builds and analyses the models. Combining modelling approaches can support this negotiation of what is important and what the implications are, but it has to be geared to the right questions. An integrative approach that incorporates perspectives on process and pattern, and is rooted in a local, participatory analysis, can, we argue, help build both better knowledge and understanding, but also greater authority and legitimacy for modelling efforts as they engage in the political and social realms of public health response and policy.

## 6. Conclusion

Modelling of zoonotic disease dynamics is a complex task. There is no perfect model and attempting to capture everything is impossible. Highly complex models are difficult to parameterize and validate, and may be unstable, as well as subject to over-interpretation. Instead we argue for an integrative approach that allows conversations between simpler models, recognized as limited, but contributing to an open debate about uncertainties, assumptions and interpretations. As we show, there have been important advances in modelling practice in recent years, with the linking of mechanistic process-based approaches with macroecological pattern-based modelling. We have highlighted, for example, the environmental-mechanistic framework that offers an exciting way forward, and illustrated this with an example of a Lassa fever virus model. But even this more sophisticated approach is insufficient. Many of the issues highlighted in the previous sections were not incorporated, and question some central assumptions. In this paper we argue for an integrated One Health approach that combines different approaches—process, pattern and participatory—not in a single model, but in a productive conversation that challenges assumptions, provides new data and offers deeper insights into the complex ‘black box’ of disease transmission.

Where knowledge is limited and systems are complex and fast-changing this must involve insights from local contexts. Involving local people in the process is vital, as only then can the contextual dynamics be properly understood. This has to be combined with a historically informed political ecology analysis that gets to grips with the underlying causal processes. This requires a deeper, denser social knowledge of who moves where, who is related to whom, who does what and why—and who gets sick through what processes. This is not easy information to get, as it may be hidden and sensitive, but any modelling effort that fails to grapple with such questions, and makes too many assumptions about, for example, random interactions, uniform behaviours or even spread, will fail—and potentially mislead. Modelling efforts for disease prediction, preparedness and response are, we argue, made more robust by the engagement with local contexts and knowledges.

We argue in particular that participatory modelling efforts can help in structuring models, defining parameters, providing data and testing model reliability. The aim must not be to try and model everything, aiming for ever greater accuracy, and more and more specification, with more data requirements. Different modelling efforts—whether process-based, pattern-based or participatory, or combinations of each—should continue to deepen and extend their approaches, but the crucial aspect is the conversation and negotiation between them. This must be open, honest and frank, and embrace uncertainties and challenge assumptions. This allows us to ask the right questions, test results, and push different modelling approaches, so outputs are more robust, geared to the appropriate scale, and responsive to social and ecological conditions. The result will be a more targeted and effective response that is inevitably plural and conditional given intersecting uncertainties and complexities [119]. Rooted in a more inclusive, co-produced process, this can provide new forms of legitimacy and authority for modelling efforts in disease outbreak contexts, and so more effective governance of zoonotic disease.
